# Supplementary material for: A novel Cep120-dependent mechanism inhibits centriole maturation in quiescent cells
Source: eLife. 2018 May 9;7:e35439. doi: 10.7554/eLife.35439 (PMC5986273; doi:10.7554/eLife.35439)
Supplement: Supplementary file 1. [file elife-35439-supp1.docx]

| **Antibody (clone #):** | **Manufacturer / (Catalog #):** | **Dilution/Fixative for IF:** | **Immunoblotting:** |
| --- | --- | --- | --- |
| Rat anti-Cep120 (Rat #02) | This study | 1:2,000/MeOH | 1: 5,000-1:10,000 |
| Mouse anti-Actin (clone AC-40) | SIGMA - A4700 | 1:200/ MeOH | 1:3,000 |
| Rabbit anti-AZI1 | Abcam - ab84864 | 1:300/(1:1MeOH /Acet.) |  |
| Rat anti-α-Tubulin (clone YL1/2) | Santa Cruz - sc-53029 | 1:200/MeOH |  |
| Mouse anti-α-Tubulin  (clone DM1A) | SIGMA - T6199 | 1:4,000/4%PFA |  |
| Mouse anti-acetylated α-Tubulin (clone 6-11B-1) | SIGMA - T6793 | 1:10,000/MeOH |  |
| Mouse anti-γ-Tubulin (clone GTU-88) | SIGMA - T6557 | 1:500-1:1,000/MeOH | 1:10,000 |
| Mouse anti-glutamylated Tubulin (GT335) | AdipoGen - AG-20B-0020 | 1:10,000/MeOH |  |
| Rabbit anti-CDK5Rap2 | Bethyl Labs - IHC-00063 | 1:500/MeOH |  |
| Rabbit anti-CDK5Rap2 | Bethyl Labs - A300-554 |  | 1:500 |
| Mouse anti-Centrin (clone 20H5) | EMD Millipore - 04-1624 | 1:1,000/MeOH/2%PFA |  |
| Mouse anti-Cep170 (clone 72-413-1) | Invitrogen - 41-3200 | 1:500/MeOH/2%PFA | 1:2,000 |
| Rabbit anti-Cep164 | (Firat-Karalar et al., 2014b) | 1:1000/MeOH |  |
| Rabbit anti-Cep290 | Abcam - ab84870 | 1:1000/MeOH |  |
| Guinea Pig anti-Ccdc11 (GP549) | (Silva et al., 2016) |  | 1:1,000 |
| Rabbit anti-Ccdc11 | (Silva et al., 2016) | 1:250/MeOH |  |
| Rabbit anti-Cp110 | (Chang et al., 2010) | 1:200/MeOH* |  |
| Mouse anti-EB1 (clone 5) | BD Biosciences - 610534 | 1:100/MeOH |  |
| Rabbit anti-FBF1 | SIGMA - HPA023677 | 1:200/MeOH* |  |
| Chicken ant-GFP (IgY) | Life Technologies – A10262 | 1:500/MeOH |  |
| Rabbit anti-IFT88 | Proteintech - 13967-1-AP | 1:150 ON/MeOH |  |
| Rabbit anti-Ki-67 | Novocastra - NCL-Ki67p | 1:1,000/MeOH |  |
| Mouse anti-Myc (clone 4A6) | EMD Millipore - 05-724 | 1:500/MeOH |  |
| Rabbit anti-Ninein | (Sillibourne et al., 2010) | 1:5,000-1:10,000/MeOH | 1:1,000 |
| Mouse anti-p150[Glued] (Clone 1) | BD Biosciences - 610473 | 1:1,000/ MeOH/4%PFA# | 1:1,000 |
| Rabbit anti-Odf2 | SIGMA - HPA001874 | 1:200/MeOH* |  |
| Rabbit anti-Ofd1 | (Tang et al., 2013) | 1:2,000/MeOH* |  |
| Rabbit anti-PCM1 (clone H-262) | Santa Cruz - sc-67204 |  | 1:200 |
| Rabbit anti-PCM1 | (Firat-Karalar et al., 2014a) | 1:3,000/MeOH |  |
| Rabbit anti-Pericentrin | Abcam - ab4448 | 1:5,000/MeOH | 1:1,000 |
| Rabbit anti-Smoothened | Abcam - ab38686 | 1:250 ON/4%PFA |  |

**Supplementary File 1 –** List of antibodies used in this study­­.

(#) - Pre-extract in buffer (20mM PIPES, pH 6.8; 0.2% Triton X-100, 10 mM EGTA, 1 mM Mg Cl_2_) for 2 min.

(*) Pre-extract in PBS supplemented with 0.1% Triton-X for 30 sec.

(ON) - Overnight incubation at 4°C
