# Supplementary material for: A novel Cep120-dependent mechanism inhibits centriole maturation in quiescent cells
Source: eLife. 2018 May 9;7:e35439. doi: 10.7554/eLife.35439 (PMC5986273; doi:10.7554/eLife.35439)
Supplement: Transparent reporting form [file elife-35439-transrepform.pdf]

1. What background information would someone who is completely unfamiliar with your field need to know to understand the findings in your paper? (Suggested word limit: 150 words)

The centrosome is the major microtubule-organizing center in animal cells, which regulates the assembly of the interphase microtubule array and mitotic spindle. Almost all cells in the human body contain a single centrosome. At the core of the centrosome are two cylindrical microtubule-based structures called centrioles. In quiescent cells, these two centrioles are different with regards to their age, morphology and function. The older of the two centrioles was assembled at least two cell cycles ago and is commonly called the “mother” centriole. The younger one, which was assembled in the previous cell cycle, is called the “daughter” centriole.

2. What exact research question did you set out to answer and why? (Suggested word limit: 75 words)

Most activities at the centrosome are controlled and mediated by the mother centriole. For example, the mother centriole contains protein complexes called appendages that give it the ability to dock at the plasma membrane and build a cilium, an important chemosensory organelle in cells. The mother centriole also contains a complex of proteins called the pericentriolar material, which helps it nucleate and anchor microtubules. In contrast, the daughter centriole lacks these appendages, does not form a cilium, and possesses significantly less pericentriolar material and microtubule nucleating ability. How and why the daughter centriole is prohibited from recruiting these protein complexes remains a mystery.

3. What are the most important findings of your paper? (Suggested word limit: 100 words)

Here, Betleja and colleagues study the role of a protein, Cep120, in establishing the differences between mother and daughter centrioles during quiescence. Using mouse and human cells, they show that Cep120 plays an important inhibitory role at the daughter centriole. Loss of Cep120 causes an increase in the amount pericentriolar material that associates with the daughter centriole, and changes the microtubule nucleation activities at the centrosome. The loss of Cep120 causes defective protein trafficking to the centrosome, which ultimately disrupts ciliary assembly and signaling.

4. Who might eventually benefit from the findings of your study, and what would need to be done before we could achieve these benefits? (Suggested word limit: 75 words)

Betleja et al’s findings show that there is a Cep120-dependent mechanism that actively regulates centrosome homeostasis in quiescent cells. These experiments uncover a potentially new type of molecular defect that may underlie the pathogenesis of ciliopathies, diseases caused by defective cilia. The next challenge will be to understand how Cep120 performs its inhibitory functions specifically at the daughter centriole.
